# Chloride Ions Are Required for Thermosipho africanus MurJ Function

**DOI:** 10.1128/mbio.00089-23

**Published:** 2023-02-08

**Authors:** Sujeet Kumar, Aurelio Mollo, Frederick A. Rubino, Daniel Kahne, Natividad Ruiz

**Affiliations:** a Department of Microbiology, The Ohio State University, Columbus, Ohio, USA; b Department of Chemistry and Chemical Biology, Harvard University, Cambridge, Massachusetts, USA; c Department of Molecular and Cellular Biology, Harvard University, Cambridge, Massachusetts, USA; d Department of Biological Chemistry and Molecular Pharmacology, Harvard Medical School, Boston, Massachusetts, USA; Fred Hutchinson Cancer Center

**Keywords:** peptidoglycan, lipid II, cell wall, membrane transporter, glycolipid, chloride ion, alternating access, counterion, flippase, lipid transport

## Abstract

Most bacteria have a peptidoglycan cell wall that determines their cell shape and helps them resist osmotic lysis. Peptidoglycan synthesis depends on the translocation of the lipid-linked precursor lipid II across the cytoplasmic membrane by the MurJ flippase. Structure-function analyses of MurJ from Thermosipho africanus (MurJ_Ta_) and Escherichia coli (MurJ_Ec_) have revealed that MurJ adopts multiple conformations and utilizes an alternating-access mechanism to flip lipid II. MurJ_Ec_ activity relies on membrane potential, but the specific counterion has not been identified. Crystal structures of MurJ_Ta_ revealed a chloride ion bound to the N-lobe of the flippase and a sodium ion in its C-lobe, but the role of these ions in transport is unknown. Here, we investigated the effect of various ions on the function of MurJ_Ta_ and MurJ_Ec_ *in vivo*. We found that chloride, and not sodium, ions are necessary for MurJ_Ta_ function, but neither ion is required for MurJ_Ec_ function. We also showed that *murJ_Ta_* alleles encoding changes at the crystallographically identified sodium-binding site still complement the loss of native *murJ_Ec_*, although they decreased protein stability and/or function. Based on our data and previous work, we propose that chloride ions are necessary for the conformational change that resets MurJ_Ta_ after lipid II translocation and suggest that MurJ orthologs may function similarly but differ in their requirements for counterions.

## OBSERVATION

**FIG 1:**
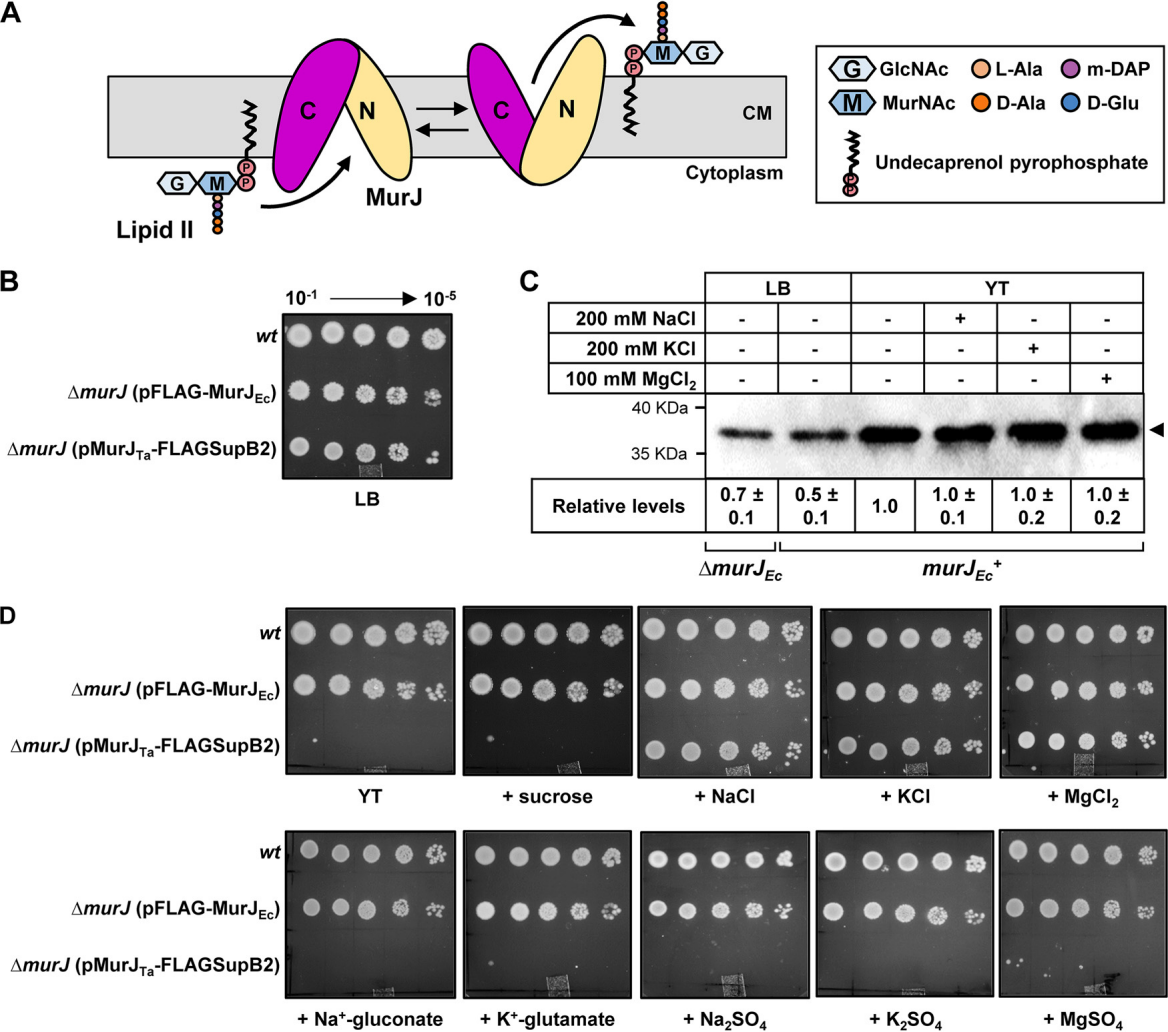
Chloride ions are required for MurJ_Ta_ function. (A) Schematic of lipid II transport by MurJ. The lipid-linked peptidoglycan precursor, lipid II, is synthesized on the cytoplasmic side of the cytoplasmic membrane (CM) and flipped by MurJ for subsequent polymerization into the peptidoglycan cell wall (not shown). MurJ’s N lobe (TMs 1 to 6) and C lobe (TMs 7 to 12) are colored gold and purple, respectively. TMs 13 to 14 are not shown for simplicity. (B) Plasmid-encoded MurJ_Ta_ complements growth of a Δ*murJ* E. coli strain on LB agar as shown by spot dilution assay. Wild-type strain NR754 (wt) carrying native *murJ* and haploid strains NR5191 (Δ*murJ*::*FRT murJ_Ec_*) and NR5996 (Δ*murJ*::*FRT murJ_Ta_*) were grown logarithmically in LB, normalized to the cell density of NR754, and serially diluted 10-fold before spot plating as described in Text S1 in the supplemental material. The plate was grown overnight at 37°C for 24 h. The pMurJTa-FLAGSupB2 plasmid contains a suppressor mutation in the promoter region that both allows complementation in the absence of IPTG and overcomes the IPTG-dependent lethality observed by overproduction of MurJ_Ta_ (see Text S1 for more details). (C) Relative levels of MurJ_Ta_ in haploid (*ΔmurJ_Ec_*) and merodiploid (*murJ_Ec_^+^*) strains were determined using FLAG immunoblotting from whole-cell lysate samples prepared from overnight cultures under different conditions. MurJ_Ta_-FLAG levels are higher in cells grown in YT medium than in LB for unknown reasons. MurJ_Ta_-FLAG levels are similar in YT media in the absence and presence of various chloride salts. The intensity of the signal from the MurJ_Ta_-FLAG band was measured, and values shown below the immunoblot denote the average ± standard deviation for three biological replicates of the signal in each sample relative to that of the sample from the strain grown in YT media (value from YT sample was set 1). (D) Spot assays showing that, unlike MurJ_Ec_, MurJ_Ta_-FLAG cannot complement the loss of native *murJ* in E. coli on YT agar unless chloride ions are added. The assay was done as in panel B except that cells (grown overnight in LB) were washed twice with YT medium before dilution. Salts and sucrose were added to a final concentration of 200 mM, except for MgCl_2_, which was at 100 mM, because of toxicity observed at 200 mM in all strains.

The peptidoglycan cell wall is an essential glycopeptide matrix that most bacteria build outside their cytoplasmic membrane (1, 2). The peptidoglycan building block is synthesized in the cytoplasm and anchored to the membrane by the lipid carrier undecaprenyl phosphate (Fig. 1A). The resulting lipid-linked precursor, lipid II, is then transported across the membrane by MurJ (or unrelated alternative flippases) so that it can be used to build the peptidoglycan cell wall during cell growth and division (1–3).

10.1128/mbio.00089-23.9 TEXT S1 Supplemental materials and methods and references. Download Text S1, PDF file, 0.2 MB. Copyright © 2023 Kumar et al. 2023 Kumar et al. https://creativecommons.org/licenses/by/4.0/ This content is distributed under the terms of the Creative Commons Attribution 4.0 International license.

MurJ is a widely conserved lipid II flippase that is essential for peptidoglycan synthesis and thereby growth in Escherichia coli (1, 4–12). Structure-function and biochemical studies of MurJ from Thermosipho africanus (MurJ_Ta_) and E. coli (MurJ_Ec_) support an alternating-access mechanism for lipid II transport that depends on membrane potential (11, 13–18). The first 12 of the 14 transmembrane α-helices (TMs) of MurJ are grouped into two lobes, the N-lobe (TMs 1 to 6) and C-lobe (TMs 7 to 12). These lobes are connected by 2-fold pseudorotational symmetry and form a V-shaped central cavity enclosed by TMs 1, 2, 7, and 8 (9–11). This cavity contains three arginines (R18, R24, and R255 in MurJ_Ta_; R18, R24, and R270 in MurJ_Ec_) that are essential for function, presumably because they interact with lipid II (10, 11, 16). TMs 13 and 14 form a hydrophobic groove that is connected to the cavity via a membrane portal flanked by TMs 1 and 8. It has been postulated that, during transport, this hydrophobic groove interacts with the undecaprenyl tail of lipid II, whereas the pyrophosphate-disaccharide-pentapeptide resides in the hydrophilic central cavity (1, 9). Crystal structures capturing MurJ_Ta_ in multiple conformations support a model where the inward-open cavity binds lipid II and transitions to an outward-open state to deliver its cargo and later reset to its inward-open state (Fig. 1A) (10).

The mechanism of energy coupling in MurJ remains unknown. Membrane potential, and not the proton gradient across the membrane, is required for MurJ_Ec_ function; data suggest that MurJ_Ec_ imports a counter ion after lipid II translocation to restart the transport cycle (1, 8, 13). Inward-facing and inward-closed structures of MurJ_Ta_ captured a chloride ion in the N-lobe that is coordinated by tyrosine Y41 and the essential arginines R24 and R255 (see Table S1 in the supplemental material), which are proposed to be part of the substrate-binding site (1, 9, 10, 14, 16). It has therefore been suggested that this chloride ion may trigger the resetting from the outward-open to the inward-open state after lipid II flipping (1, 9). Interestingly, molecular dynamics simulations and structural alignments with MurJ_Ta_ of a related drug exporter, PfMATE, showed the spontaneous and specific binding of a chloride ion to the inward-open structure of PfMATE at the equivalent location (1, 9, 19). In addition, a sodium ion was found in the C-lobe of outward-facing, inward-occluded, and inward-closed MurJ_Ta_ structures (10). The sodium ion is held in position by trigonal bipyramidal coordination created by residues D235, N374, D378, V390, and T394, which are not conserved in MurJ_Ec_ (9, 10, 18). Unfortunately, the functional relevance of these sodium-binding residues could not be determined because changing them through mutagenesis caused a loss of MurJ production and/or its degradation (10). Surprisingly, neither chloride nor sodium ions have been found in MurJ_Ec_ structures so far (11, 18).

10.1128/mbio.00089-23.6 TABLE S1 Conservation of residues implicated in binding to chloride ions in MurJ_Ta_. Download Table S1, PDF file, 0.2 MB. Copyright © 2023 Kumar et al. 2023 Kumar et al. https://creativecommons.org/licenses/by/4.0/ This content is distributed under the terms of the Creative Commons Attribution 4.0 International license.

Here, we investigated the role of chloride and sodium ions in MurJ_Ta_ and MurJ_Ec_ by exploiting the fact that plasmid-encoded MurJ_Ta_ has been shown to function in Escherichia coli (14). We first altered a previously published plasmid encoding FLAG-tagged MurJ_Ec_ to instead encode MurJ_Ta_-FLAG. The resulting plasmid-encoded MurJ_Ta_-FLAG supported growth of E. coli cells lacking chromosomal *murJ* on LB agar (Fig. 1B; for more details, see Text S1, Table S2 and S3) and was readily detected through immunoblotting (Fig. 1C). Since LB contains NaCl, we tested the growth of the haploid strains carrying either *murJ_Ta_* or *murJ_Ec_* on YT (i.e., LB without NaCl) or glucose M63 minimal medium agar (which lacks NaCl), supplemented or not with different salts or the osmolyte sucrose. While the *murJ_Ec_* haploid strain grew on yeast extract-tryptone (YT) or glucose M63 agar without added salts or sucrose, the *murJ_Ta_* haploid strain only grew when the YT or M63 agar was supplemented with chloride salts, even in the absence of sodium (Fig. 1D and Fig. S1A). The presence or absence of chloride or sodium in YT agar did not affect MurJ_Ta_ levels in merodiploid cells expressing both the native chromosomal *murJ_Ec_* and plasmid-borne *murJ_Ta_*, indicating that the loss of viability of the haploid strain on YT when we did not add these ions was not caused by defects in synthesis or folding of MurJ_Ta_ (Fig. 1C). However, as previously reported for MurJ_Ec_ produced from the same plasmid backbone, we could not detect MurJ_Ta_ in samples from cells grown in M63 medium regardless of the salts present in the absence of IPTG (Fig. S1B) (16). Notably, overexpression of *murJ_Ta_* with IPTG (isopropyl-β-d-thiogalactopyranoside) did not rescue growth on either chloride-free YT or glucose M63 medium (Fig. S1C). Together, our findings indicate that chloride ions, rather than sodium ions, are essential for the function of MurJ_Ta_, but not MurJ_Ec_. Thus, the essential R24 and R255 residues in MurJ_Ta_ might be required for both lipid II and chloride binding in different steps of the transport cycle (Table S1).

10.1128/mbio.00089-23.1 FIG S1 Chloride is specifically required for MurJ_Ta_-dependent growth on M63 minimal agar. (A) Spot dilution assay for NR754 (wt) and haploid E. coli strains complemented with *murJ_Ec_* (NR5191) or *murJ_Ta_* (NR5996) on glucose M63 agar plates with or without sucrose and various salts. Strains were grown exponentially in LB, normalized to the cell density of NR754, washed twice, serially diluted 10-fold in M63 medium, and spotted on glucose M63 agar plates, which were incubated at 37°C for 24 h. Salts and sucrose were added to a final concentration of 200 mM except for MgCl_2_, which was added at 100 mM because of toxicity at higher concentrations. The haploid strain complemented with MurJ_Ec_ grows on all M63 plates, but the haploid strain complemented with MurJ_Ta_ only grows on M63 media supplemented with chloride salts. (B) Relative levels of MurJ_Ta_ in merodiploid strains carrying chromosomal *murJ_Ec_* were determined using FLAG immunoblotting of whole-cell lysate samples prepared from overnight cultures grown in LB or glucose M63 medium. MurJ_Ta_ levels are not detected in M63 medium as previously reported for MurJ_Ec_ produced from the same plasmid backbone. (C) Spot dilution assay for NR754 (wt), NR5996 (Δ*murJ*::*FRT*, *murJ_Ta_*), and NR6219 (*murJ_Ec_^+^ murJ_Ta_*) on various agar plates with or without 50 μM IPTG. Overexpression of *murJ_Ta_* with IPTG did not rescue growth on chloride-free YT or M63 plates. (D) Relative levels of plasmid-encoded MurJ_Ta_ in the merodiploid *murJ_Ec_^+^* (NR6219) and haploid *ΔmurJ* (NR5996) strains were determined using FLAG immunoblotting of whole-cell lysate samples prepared from overnight cultures grown in LB, YT, or glucose M63 in the presence or absence of IPTG. The immunoblot analysis showed that MurJ_Ta_ levels are increased in presence of IPTG in all growth media. Data shown are representative of three biological replicates. Download FIG S1, PDF file, 1.1 MB. Copyright © 2023 Kumar et al. 2023 Kumar et al. https://creativecommons.org/licenses/by/4.0/ This content is distributed under the terms of the Creative Commons Attribution 4.0 International license.

Some chloride transporters have been shown to also function with bromide and iodide (but not fluoride) ions, which belong to the same halogen group as chloride (20–22). We therefore tested the effect of bromide, iodide, and fluoride ions on MurJ_Ta_. We found that MurJ_Ta_ could support growth of E. coli in the presence of the larger bromide and iodide ions but not when YT or M63 was supplemented with the smaller fluoride ion (Fig. S4). In light of these findings, we changed the crystallographically recognized chloride-binding site in MurJ_Ta_ by making variants of the ion-coordinating residue Y41 and evaluating their ability to compensate for the loss of native *murJ* in E. coli (note that the arginines in the binding site are essential) (10). We substituted Y41 to phenylalanine since it is the equivalent residue in MurJ_Ec_ and to alanine to drastically change its side chain. We found that plasmid-encoded MurJ_Ta_^Y41F^ behaved like wild-type MurJ_Ta_; however, MurJ_Ta_^Y41A^ supported growth on LB plates only in the presence of IPTG and required a higher concentration of Cl^−^ salt to support growth on YT and M63 media in the absence of IPTG. These results suggested that reducing the size of chloride-binding pocket might affect the affinity toward the chloride ion (Fig. S5). We also observed that while the Y41F substitution led to increased levels of MurJ_Ta_, the Y41A substitution did not alter MurJ_Ta_ levels (Fig. S5C). Furthermore, we tested if MurJ_Ta_ and MurJ_Ec_ differ in their requirement for chloride because they have a different amino acid at this position (tyrosine in MurJ_Ta_ and phenylalanine in MurJ_Ec_), but we found that a MurJ_Ec_^F41Y^ variant behaved like wild-type MurJ_Ec_, and its function did not become chloride dependent (Fig. S5). Thus, we still do not understand why MurJ_Ec_ does not require the addition of chloride ions.

10.1128/mbio.00089-23.4 FIG S4 Bromide (Br^−^) and iodide (I^−^), but not fluoride (F^−^), can support MurJ_Ta_ function. (A, B) Wild-type strain NR754 (wt) carrying native *murJ* and haploid strains NR5191 (Δ*murJ*::*FRT murJ_Ec_*) and NR5996 (Δ*murJ*::*FRT murJ_Ta_*) were grown logarithmically in LB, normalized to the cell density of NR754, and serially diluted 10-fold before spot plating as described in Text S1. Plates were grown overnight at 37°C for 24 h. NaBr and NaCl salts were added to a final concentration of 200 mM. Strains exhibited growth defects to various extents in the presence of 100 and 200 mM NaF and KI. Results indicate that Br^−^ and I^−^ ions (like Cl^−^ ions) can support MurJ_Ta_-FLAG function, but F^−^ ions cannot. (C) Spot assay was done as described above but using LB supplemented with 50 and 100 mM NaF. Download FIG S4, PDF file, 1.0 MB. Copyright © 2023 Kumar et al. 2023 Kumar et al. https://creativecommons.org/licenses/by/4.0/ This content is distributed under the terms of the Creative Commons Attribution 4.0 International license.

10.1128/mbio.00089-23.5 FIG S5 Crystallographically identified chloride-binding site residues are important for MurJ_Ta_ function. (A, B). Spot dilution assay on various agar plates for MurJ_Ec_ and MurJ_Ta_ variants with changes in the proposed chloride-binding site were conducted as described in Text S1. Haploid Δ*murJ::FRT* E. coli strains complemented with plasmids encoding wild-type FLAG-MurJ_Ec_ (strain NR5191), MurJ_Ta_-FLAG (strain NR5996), or variants with changes, FLAG-MurJ_Ec_/F41Y (strain NR7739), MurJ_Ta_-FLAG/Y41F (strain NR7740), and MurJ_Ta_-FLAG/Y41A (strain NR7748), were grown exponentially in LB with 50 μM IPTG (except for NR5191 and NR7739 due to toxicity in presence of IPTG) and normalized to the cell density of the wild-type strain. Cells were washed twice in YT and serially diluted 10-fold before spotting on agar plates, which were incubated at 37°C for 24 h. Strains producing FLAG-MurJ_Ec_/F41Y and MurJ_Ta_-FLAG/Y41F variants behaved like their respective wild-type parent strains. The strain producing the MurJ_Ta_-FLAG/Y41A variant showed growth defect in the absence of IPTG on LB and required a higher concentration of chloride salts (>200 mM) for growth on YT and M63 medium. (C) FLAG immunoblotting showing the relative levels of FLAG-MurJ_Ec_ and MurJ_Ta_-FLAG (wild-type or chloride-site variants) in whole-cell samples from haploid Δ*murJ* E. coli strains grown overnight in LB with or without 50 μM IPTG. Results showed that the F41Y and Y41F substitutions cause an increase in MurJ_Ec_ and MurJ_Ta_ levels, respectively. Data shown are representative of three biological replicates. Download FIG S5, PDF file, 1.3 MB. Copyright © 2023 Kumar et al. 2023 Kumar et al. https://creativecommons.org/licenses/by/4.0/ This content is distributed under the terms of the Creative Commons Attribution 4.0 International license.

**FIG 2:**
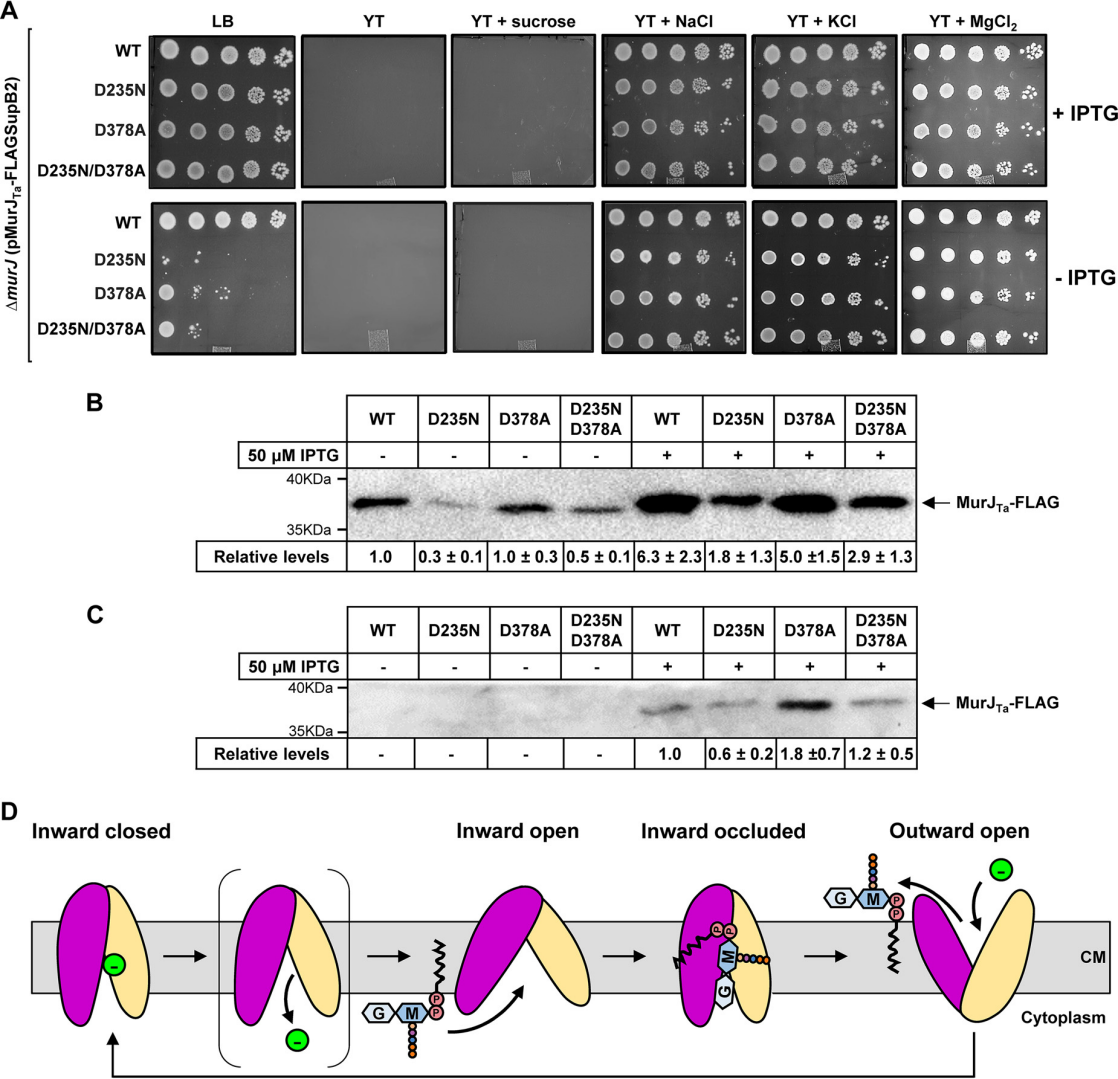
Crystallographically identified sodium-binding site residues are not essential for MurJ_Ta_ function. (A) Spot dilution assay on various agar plates for MurJ_Ta_ variants with changes in the sodium-binding site conducted as in Fig. 1. Haploid Δ*murJ::FRT* E. coli strains complemented with plasmids encoding wild-type (labeled WT), MurJ_Ta_-FLAG (strain NR5996), or variants with changes, D235N (strain NR7491), D378A (strain NR7492), or both (strain NR7493), were grown exponentially in LB with 50 μM IPTG and normalized to the cell density of the wild-type strain NR5996. Cells were washed twice in YT and serially diluted 10-fold before spotting on agar plates, which were incubated at 37°C for 24 h. The sodium-binding site mutants showed growth defects in the absence of IPTG on LB, and, like their wild-type parent, required the addition of chloride salts for growth on YT medium. (B) FLAG immunoblotting was used to determine the relative levels of MurJ_Ta_-FLAG (wild-type or sodium-site variants) in whole-cell samples from haploid Δ*murJ* E. coli strains grown overnight in LB with or without 50 μM IPTG. The immunoblot analysis revealed that the D235N change causes a decrease in MurJ_Ta_ levels. Values shown below the immunoblot denote average ± standard deviation for three biological replicates of the signal in each sample relative to that of the WT sample (the value for WT was set to 1.0). (C) FLAG immunoblotting showing the relative levels of MurJ_Ta_ (wild-type or sodium-site variants) in whole-cell samples prepared from merodiploid strains of E. coli carrying the native chromosomal *murJ* allele grown overnight in glucose M63 with or without 50 μM IPTG. The MurJ_Ta_ variants are only detected in the presence of IPTG. (D) Updated model of lipid II transport by MurJ_Ta._ Inward-open MurJ binds to lipid II to adopt an inward-occluded state in which the pyrophosphate-disaccharide-pentapeptide moiety resides in the hydrophilic central cavity and the undecaprenyl lipid binds to the hydrophobic groove in TMs 13 to 14 (not labeled in cartoon). The formation of the MurJ-lipid II complex leads to a transition to the outward-open state that releases lipid II into the outer leaflet of the membrane and binds to chloride to reset MurJ_Ta_ to the inward-closed state. A new transport cycle starts when the inward-closed state releases the chloride ion to transition into the inward-open state after going through a putative intermediate shown in brackets.

Considering these results, we reexamined the importance of the crystallographically identified sodium-binding site in MurJ_Ta_ by generating variants with changes in the ion-coordinating residues D235 and D378 and characterizing their ability to complement the loss of native *murJ* in E. coli. In a previous study, MurJ_Ta_^D235N^ and MurJ_Ta_^D378A^ produced from a plasmid different than the one used here were unable to complement in E. coli, and the proteins were undetectable, although a MurJ_Ta_^D378N^ variant complemented (10). Using our plasmid system, we found that plasmid-encoded MurJ_Ta_^D235N^, MurJ_Ta_^D378A^, and MurJ_Ta_^D235N/D378A^ complemented the loss of chromosomal *murJ* in E. coli on LB agar in the presence of IPTG (Fig. 2A). However, unlike wild-type MurJ_Ta_, these variants could not fully support growth on LB agar in the absence of IPTG (Fig. 2A and Fig. S2A). As previously reported, the D235N substitution led to reduced levels of MurJ_Ta_, but the D378A substitution did not (Fig. 2B and C and Fig. S2B and C). We found that, like those carrying the wild-type *murJ_Ta_* allele, haploid strains carrying the mutant alleles required the presence of chloride, not sodium, ions to grow on YT or M63 agar (Fig. 2A, Fig. S2A, and Fig. S3). The addition of chloride was sufficient to restore growth in these media even in the absence of IPTG, likely because of the higher level of MurJ_Ta_ produced in YT and the lower growth rate in M63 minimal medium (Fig. 2A, Fig. S2A, and Fig. S3). We could also restore growth on LB agar of haploid strains producing either MurJ_Ta_^D378A^ or MurJ_Ta_^D235N/D378A^ to near wild-type level by adding twice as much NaCl (LB Miller) (Fig. 2SA). Thus, these data show that the side chain of residue D235 is structurally (and likely functionally) important, while that of D378 contributes to MurJ_Ta_ function, but neither is essential for MurJ_Ta_ function. These findings agree with previous data from Kuk et al. showing that residues in the equatorial plane of the Na^+^-binding site (e.g., D235) are more critical than those located in the axial plane (e.g., D378) (10). Furthermore, it is possible that Na^+^, instead of serving as a coupling ion for lipid II translocation, might be involved in the allosteric control of MurJ_Ta_ activity and/or structural stability.

10.1128/mbio.00089-23.2 FIG S2 Overexpression or raising the concentration of chloride but not sodium promotes growth of haploid strains producing MurJ_Ta_ sodium-binding site variants. (A) Spot dilution assay on various agar plates for haploid cells producing MurJ_Ta_ variants with changes in the crystallographically identified sodium-binding site. Haploid Δ*murJ*::*frt* E. coli strains complemented with plasmids encoding wild-type (labeled WT) MurJ_Ta_ (NR5996) or variants with changes, D235N (NR7491), D378A (NR7492), or both (NR7493), were grown exponentially in LB with 50 μM IPTG and normalized to the cell density of the wild-type strain NR5996. Cells were washed twice and serially diluted 10-fold before spotting on agar plates, which were incubated at 37°C for 24 h. Unlike the haploid strain producing wild-type MurJ_Ta_, the sodium-binding mutants showed growth defects in the absence of IPTG on LB Lennox agar. All strains required the addition of chloride salts for growth on YT medium. (B) FLAG immunoblotting was used to determine the relative levels of MurJ_Ta_ (wild-type or sodium-site variants) in whole-cell samples prepared from haploid Δ*murJ* E. coli strains grown overnight in LB Lennox or LB Miller with or without 50 μM IPTG. The D235N sodium-binding site variants are present at a lower level in both LB Lennox and LB Miller growth media. (C) Relative levels of MurJ_Ta_ (wild-type or sodium-site variants) in merodiploid strains as determined using FLAG immunoblotting of whole-cell lysate samples prepared from overnight cultures grown in LB or YT in the presence or absence of IPTG. Levels of wild-type and mutant MurJ_Ta_ are higher in YT medium than LB in the absence of IPTG because of unknown reasons. Data shown are representative of three biological replicates. Download FIG S2, PDF file, 0.8 MB. Copyright © 2023 Kumar et al. 2023 Kumar et al. https://creativecommons.org/licenses/by/4.0/ This content is distributed under the terms of the Creative Commons Attribution 4.0 International license.

10.1128/mbio.00089-23.3 FIG S3 Raising the concentration of chloride, but not sodium, ions promotes growth of haploid strains producing MurJ_Ta_ sodium-binding site variants on M63 medium. Spot dilution assay on various agar plates for MurJ_Ta_ variants of crystallographically identified sodium-binding site. Haploid Δ*murJ*::*frt* E. coli strains complemented with plasmids encoding wild-type (labeled WT) MurJ_Ta_ (NR5996), or variants with changes, D235N (NR7491), D378A (NR7492), or both (NR7493), were grown exponentially in LB with 50 μM IPTG and normalized to the cell density of the wild-type strain NR5996. Cells were washed twice and diluted in M63 and spotted on the specified agar plates, which were incubated at 37°C for 24 h. The sodium-binding mutants, like the strain producing wild-type MurJ_Ta_, require addition of chloride salts for growth on glucose M63 agar. Data shown are representative of three biological replicates. Download FIG S3, PDF file, 0.7 MB. Copyright © 2023 Kumar et al. 2023 Kumar et al. https://creativecommons.org/licenses/by/4.0/ This content is distributed under the terms of the Creative Commons Attribution 4.0 International license.

We propose a revised model for lipid II transport by MurJ_Ta_ where the flippase requires chloride but not sodium ions for function (Fig. 2D). The molecular basis for the difference in requirements we have observed for MurJ_Ta_ and MurJ_Ec_ remains unknown. However, MurJ_Ta_ might have evolved a requirement for high chloride concentrations since T. africanus lives in high-saline environments. We still do not understand the dependence of MurJ_Ec_ on membrane potential. We cannot rule out whether its effect is indirect or MurJ_Ec_ has evolved a higher affinity for an ion(s) that might be present at low levels in the many different environments in which E. coli grows, including YT and M63 media. Nevertheless, we hope our findings aid in the development of a long-awaited *in vitro* reconstitution system for MurJ.

10.1128/mbio.00089-23.7 TABLE S2 Strains used in this study. Download Table S2, PDF file, 0.2 MB. Copyright © 2023 Kumar et al. 2023 Kumar et al. https://creativecommons.org/licenses/by/4.0/ This content is distributed under the terms of the Creative Commons Attribution 4.0 International license.

10.1128/mbio.00089-23.8 TABLE S3 Primers used in this study. Download Table S3, PDF file, 0.09 MB. Copyright © 2023 Kumar et al. 2023 Kumar et al. https://creativecommons.org/licenses/by/4.0/ This content is distributed under the terms of the Creative Commons Attribution 4.0 International license.
