# Supplementary material for: Chloride Ions Are Required for Thermosipho africanus MurJ Function
Source: mBio. 2023 Feb 8;14(1):e00089-23. doi: 10.1128/mbio.00089-23 (PMC9973255; doi:10.1128/mbio.00089-23)
Supplement: TEXT S1 [file mbio.00089-23-s0009.pdf]

Supplemental Materials and Methods

Bacterial strains and growth conditions

Table S2 lists the strains used in this study. In lysogeny broth (LB or LB Lennox: 10 g/L tryptone, 5 g/L yeast extract and 5 g/L NaCl), cells were cultured at 37°C with aeration, and growth was measured by absorbance at 600 nm (A_{600}). To make LB agar, 15 g/L agar (BD Difco) was added to LB. To investigate the effects of various ions on MurJ function, either YT (10 g/L tryptone and 5 g/L yeast extract) or glucose M63 minimal medium [2 g/L glucose, 2.0 g/L $(NH4)_2SO_4$, 13.6 g/L KH_2PO_4, 0.5 mg/L $FeSO_4.7H_2O$, 1 mM $MgSO_4.7H_2O$ and 1 mg/L vitamin B1) were supplemented with salts and/or 15 g/L agar as indicated. Chloramphenicol (20 μg/ml), 5-bromo-4-chloro-3-indolyl β-D galactopyranoside (X-Gal; 20 μg/ml), and isopropyl β-D-1-thiogalactopyranoside (IPTG; 0.05 mM or 0.2 mM) was added to the growth medium when required. For some experiments, LB Miller (10 g/L tryptone, 5 g/L yeast extract and 10 g/L NaCl) was used as mentioned.

Plasmid construction and site-directed mutagenesis (SDM)

Table S3 lists the primers used in this study. pMurJ$_{Ta}$-FLAG was generated via a two-piece Gibson Assembly. The backbone was amplified from pFLAG-EcMurJ$^{\Delta Cys}$ (1), whereas the insert was amplified from a MurJ$_{Ta}$-containing plasmid encoding SUMO-Flag-MurJ$_{Ta}$, pFR174 (unpublished), using primers listed in Table S2. The resulting plasmid was further modified using primers oFR305-306 to insert the sequence encoding a C-terminal FLAG tag to construct pMurJ$_{Ta}$-FLAG. The pMurJ$_{Ta}$-FLAG plasmid was unable to fully support growth of haploid strain NR5895 likely because of low levels of expression, but addition of IPTG induced lethality of NR5895 strain due to overexpression of *murJ$_{Ta}$* as it happens with *murJ$_{EC}$* in the same plasmid system. To overcome the IPTG-dependent lethality, we selected for suppressors that were IPTG

resistant as previously described (2). Briefly, cells from 1 mL of a culture of NR5895 grown overnight in LB were pelleted and plated on LB agar plates supplemented with X-gal and 0.2 mM IPTG. We confirmed through linkage and sequencing that a plasmid isolated from one of the resulting IPTG-resistant colonies carried a mutation in the promoter region controlling expression of *murJ*$_{Ta}$*-flag*. The plasmid carrying this suppressor mutation was designated as pMurJ$_{Ta}$-FLAGSupB2 and shown to fully complement the growth of haploid strain NR5996 on IPTG-containing media.

Mutations resulting in amino acid substitutions in the sodium-binding site of MurJ$_{Ta}$ were introduced into pMurJ$_{Ta}$-FLAGSupB2 using primers listed in Table S2. Primers were designed as previously described (3) and synthesized by Genewiz to generate mutant alleles that encoded either the D235N or D378A changes or both. The pMurJ$_{Ta}$-FLAGSupB2 template was mixed with primers and 2x KOD Hot Start Master Mix (EMD Millipore). The PCR products were column purified and subjected to one- or two-piece Gibson assembly (for single and double mutants, respectively). NovaBlue transformants containing these plasmids were selected on LB agar containing 20 μg/ml of chloramphenicol and their plasmids were sequenced by nanopore technology via Plasmidsaurus.

Functionality test of pMurJ$_{Ta}$-FlagSupB2 derivatives

The potential of pMurJ$_{Ta}$-FlagSupB2 and its derivatives to complement the loss of native chromosomal *murJ* in *E. coli* was tested using strain NR3267 (NR754 Δ*murJ*::*frt* pRC7KanMurJ$_{Ec}$) as previously described (4). We screened for functionality of *murJ*$_{Ta}$ alleles using the single-copy-number plasmid pRC7KanMurJ$_{Ec}$, which has two key characteristics. First, pRC7KanMurJ$_{Ec}$ has a partitioning defect, which means that the two copies of replicated plasmid do not properly segregate into the two daughter cells. Consequently, in the

absence of kanamycin selection and the presence of a functional allele of *murJ*, it is quickly lost from the cell population. Because *murJ* is essential for growth, pRC7KanMurJ$_{Ec}$-deficient daughter cells of NR3267 do not survive. Since plasmid-free cells die, pRC7KanMurJ$_{Ec}$ is maintained in NR3267 populations even in the absence of kanamycin. Second, pRC7KanMurJ$_{Ec}$ encodes β-galactosidase (LacZ), making blue/white screening on X-Gal an easy way to identify maintenance (i. e. blue colonies) or loss (i. e. white colonies) of the plasmid. We transformed strain NR3267 with pMurJ$_{Ta}$-FlagSupB2 and its derivatives to make use of these features of pRC7KanMurJ$_{Ec}$ to assess functional complementation. Plasmids containing functional *murJ$_{Ta}$* allele produced white colonies, while nonfunctional *murJ$_{Ta}$* allele produced persistently blue colonies.

Spot-dilution plate assay

After growing overnight in LB at 37°C, *E. coli* cultures were diluted 1:100 in LB with chloramphenicol and grown to an OD_{600} of 0.7-1 in a 37°C incubator. For experiments with MurJ$_{Ta}$ sodium-binding variants, 50 μM IPTG was added to the growth media. Bacterial cells were normalized as per OD_{600} with respect to their wild-type strain and pelleted in a microcentrifuge tube. Normalized cells were washed twice in YT or M63 medium and resuspended in the same medium. Washed cells were serially diluted ten-fold, and 5 μl of 10^{-1} to 10^{-5} dilutions were spotted on LB, YT or glucose M63 agar plates containing salts or osmolytes as indicated. After 24 h at 37°C, plates were imaged. Each experiment was carried out at least three times.

FLAG immunoblotting for MurJ$_{Ta}$-FLAG detection

Samples were prepared as described previously with some modifications (4). Cells were grown overnight, normalized by dividing 800 by OD_{600} values of the cultures, pelleted by centrifugation

at 16,873 × g for 1 min at room temperature, and lysed with 50 µl BugBuster protein extraction reagent (Novagen) and 12.5 units of Benzonase (Novagen). After incubating samples for 20 min on a rotator at room temperature, 50 µl of 2× AB buffer (4) was added and the sample was heated at 45 °C for 30 min. For electrophoresis, samples were loaded onto a 10% SDS-polyacrylamide gel. Using semidry transfer equipment (Bio-Rad), proteins were transferred from the gel to a polyvinylidene difluoride (PVDF) membrane at 10 V for 2 h. The PVDF membrane was probed using anti-FLAG M2 (1:10,000; Sigma-Aldrich) and anti-mouse horseradish peroxidase (1:10,000; GE Healthcare) antibodies. The Clarity Max Western ECL substrate (Bio-Rad) was applied to develop the signal, which was detected with a ChemiDoc XRS+ system following the manufacturer's guidelines (Bio-Rad).

Supplemental Material and Methods References

1. Butler EK, Davis RM, Bari V, Nicholson PA, Ruiz N. 2013. Structure-function analysis of MurJ reveals a solvent-exposed cavity containing residues essential for peptidoglycan biogenesis in *Escherichia coli*. J Bacteriol 195:4639-49.
2. Ruiz N. 2009. *Streptococcus pyogenes* YtgP (Spy_0390) complements *Escherichia coli* strains depleted of the putative peptidoglycan flippase MurJ. Antimicrob Agents Chemother 53:3604-5.
3. Liu H, Naismith JH. 2008. An efficient one-step site-directed deletion, insertion, single and multiple-site plasmid mutagenesis protocol. BMC Biotechnol 8:91.
4. Kumar S, Rubino FA, Mendoza AG, Ruiz N. 2019. The bacterial lipid II flippase MurJ functions by an alternating-access mechanism. J Biol Chem 294:981-990.
