# Supplementary material for: Chloride Ions Are Required for Thermosipho africanus MurJ Function
Source: mBio. 2023 Feb 8;14(1):e00089-23. doi: 10.1128/mbio.00089-23 (PMC9973255; doi:10.1128/mbio.00089-23)
Supplement: TABLE S1 [file mbio.00089-23-s0006.pdf]

Table S1. Conservation of residues implicated in binding to chloride ions in $MurJ_{Ta}$.

$MurJ_{Ta}$				$MurJ_{Ec}$					
Residues in $MurJ_{Ta}$	Consurf conservation score(1)	Kuk *et al.*, 2017(2) and 2019[b] (3)	This study	Residues in $MurJ_{Ec}$	Consurf conservation score(1)	Butler *et al.*, 2013(4) and 2014[c](5)	Zheng *et al.*, 2018[d] (6)	This study	Proposed function[e] (7, 8)
R24	9	A		R24	9	A, C, E, K	G, L, Q		Cl^- (Ta) and Lipid II (Ta and Ec) binding
F28	6			V28	6	C	L, I, F, D, A, G		Cl^- binding (Ta)[f]
Y41	9		A, F	F41	8		L, I, V, S, C, Y, L	Y	Cl^- binding (Ta)
F42	3			F42	4		I, V, L, S, C, Y		Cl^- binding (Ta)[f]
F184	8			V193	8		L, I, F, G, A, D		Cl^- binding (Ta)[f] and folding (Ec)
R255	8	A		R270	7	A, C, E, K	P, C, S, L, H		Cl^- (Ta) and Lipid II (Ta and Ec) binding

[a] Consurf conservation scores range from 1-9 with 9 being the value for highest conservation.
[b, c] Residues in **red** are total-loss-of-function variants (unable to complement the loss of wild-type MurJ in E. coli); residues in **green** refer to variants that behave like wild-type MurJEc (complements the loss of wild-type MurJ in E. coli); residue in **magenta** is a partial-loss-of-function variant (defective but complements the loss of wild-type MurJ in E. coli).
[d] Residues in **gold** are defective as determined through MutSeq by the fact that this site mutation frequency fold change was >2.5 when complementing wild-type MurJ was produced compared to when it was not; residues in **blue** are not defective as determined through MutSeq by the fact that this site mutation frequency fold change was <2.5 when complementing wild-type MurJ was produced compared to when it was not.
[e] Ta refers to $MurJ_{Ta}$; Ec refers to $MurJ_{Ec}$

[f] Residues were noted to be oriented in ways that are suitable to form anion-quadrupole interactions with chloride ion by Kuk *et al*, 2022, but their role in $MurJ_{Ta}$ was not tested by the authors or in this study.

References:

1. Ashkenazy H, Abadi S, Martz E, Chay O, Mayrose I, Pupko T, Ben-Tal N. 2016. ConSurf 2016: an improved methodology to estimate and visualize evolutionary conservation in macromolecules. Nucleic Acids Res 44:W344-50.

2. Kuk AC, Mashalidis EH, Lee SY. 2017. Crystal structure of the MOP flippase MurJ in an inward-facing conformation. Nat Struct Mol Biol 24:171-176.

3. Kuk ACY, Hao A, Guan Z, Lee SY. 2019. Visualizing conformation transitions of the Lipid II flippase MurJ. Nat Commun 10:1736.

4. Butler EK, Davis RM, Bari V, Nicholson PA, Ruiz N. 2013. Structure-function analysis of MurJ reveals a solvent-exposed cavity containing residues essential for peptidoglycan biogenesis in *Escherichia coli*. J Bacteriol 195:4639-49.

5. Butler EK, Tan WB, Joseph H, Ruiz N. 2014. Charge requirements of lipid II flippase activity in *Escherichia coli*. J Bacteriol 196:4111-9.

6. Zheng S, Sham LT, Rubino FA, Brock KP, Robins WP, Mekalanos JJ, Marks DS, Bernhardt TG, Kruse AC. 2018. Structure and mutagenic analysis of the lipid II flippase MurJ from *Escherichia coli*. Proc Natl Acad Sci U S A 115:6709-6714.

7. Kuk ACY, Hao A, Lee SY. 2022. Structure and mechanism of the lipid flippase MurJ. Annu Rev Biochem doi:10.1146/annurev-biochem-040320-105145.

8. Kumar S, Mollo A, Kahne D, Ruiz N. 2022. The bacterial cell wall: From Lipid II flipping to polymerization. Chem Rev doi:10.1021/acs.chemrev.1c00773.
