# Supplementary figures and images for: Chloride Ions Are Required for Thermosipho africanus MurJ Function
Source: mBio. 2023 Feb 8;14(1):e00089-23. doi: 10.1128/mbio.00089-23 (PMC9973255; doi:10.1128/mbio.00089-23)

**A**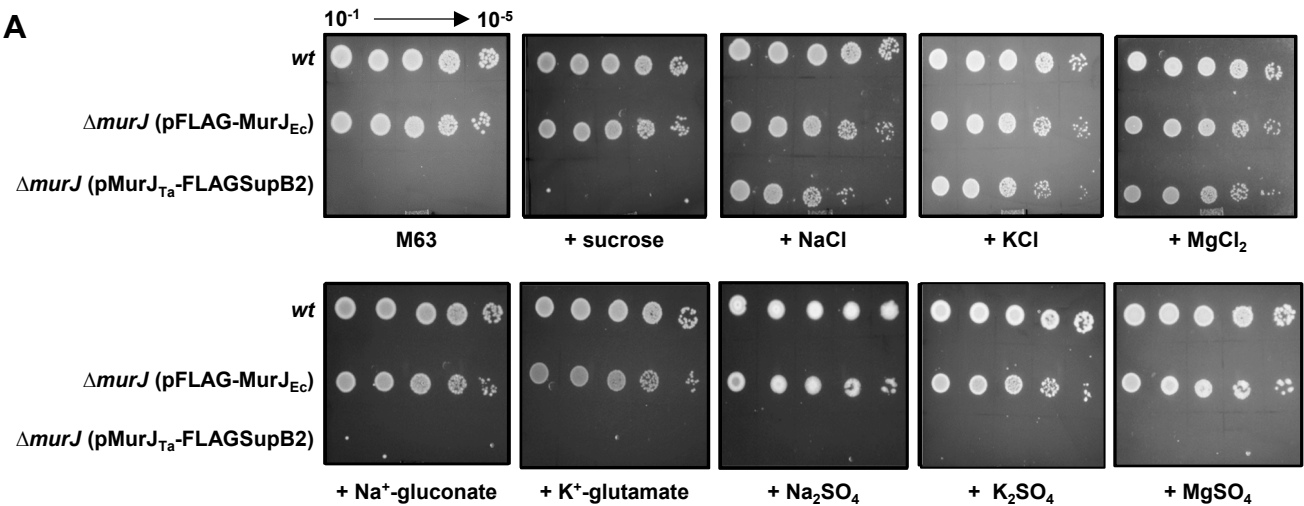**B**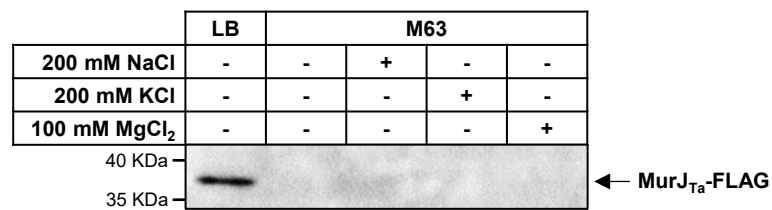**C**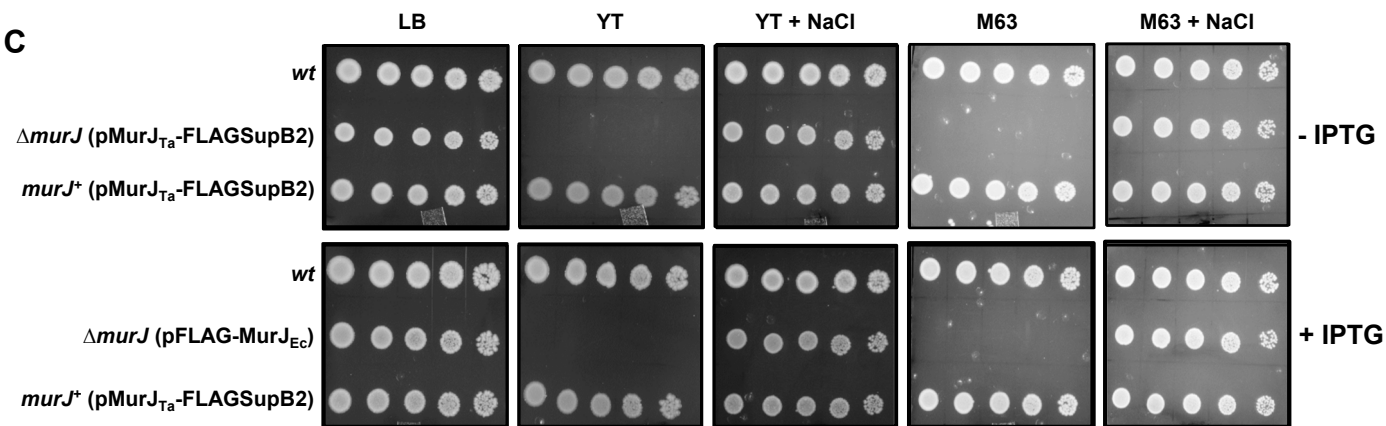**D**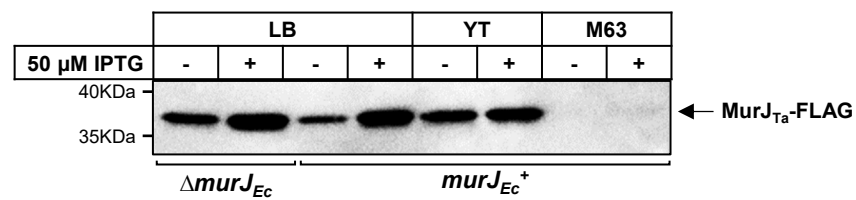

Supplement: FIG S1 [file mbio.00089-23-s0001.pdf]

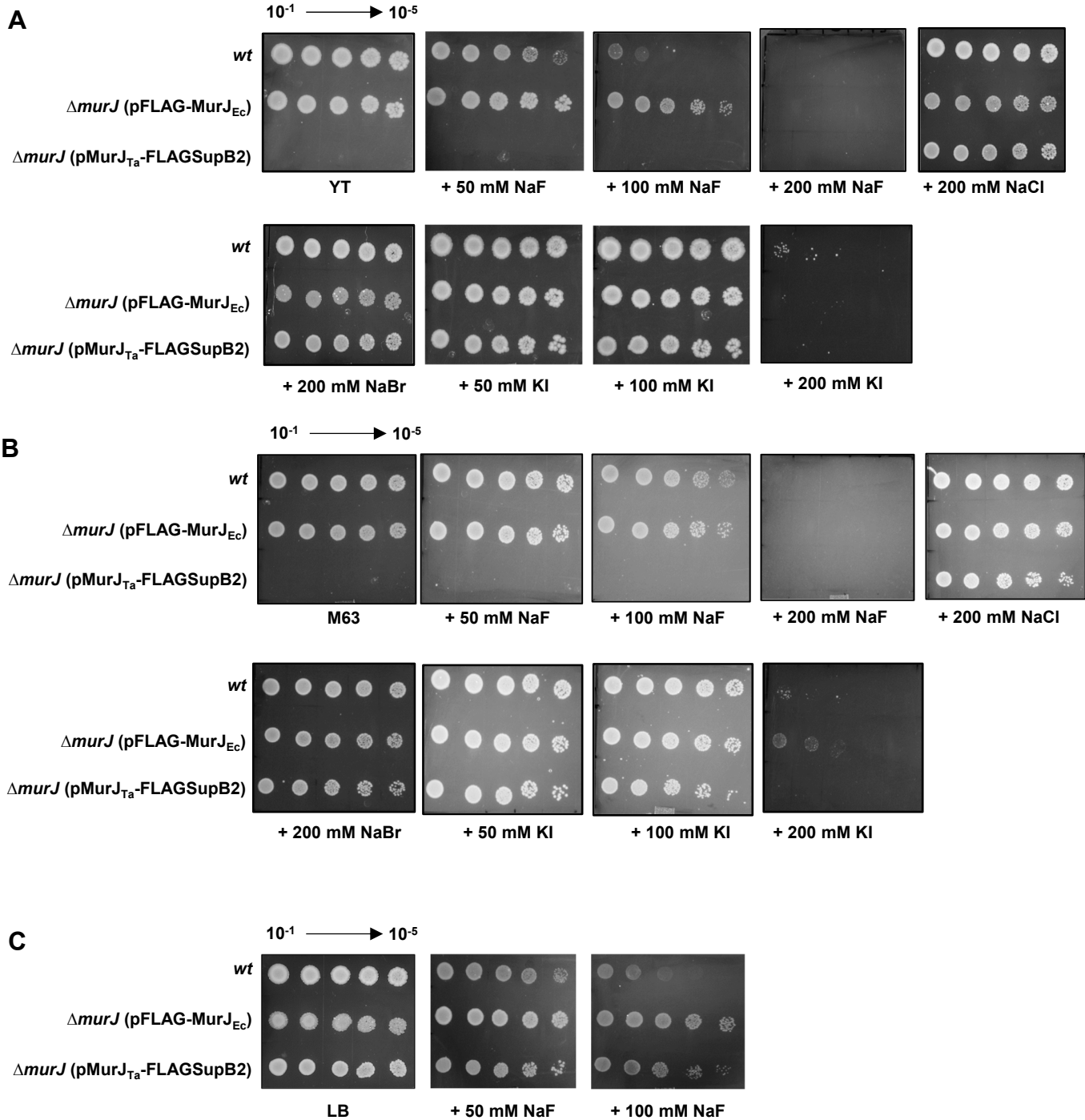

Supplement: FIG S4 [file mbio.00089-23-s0004.pdf]

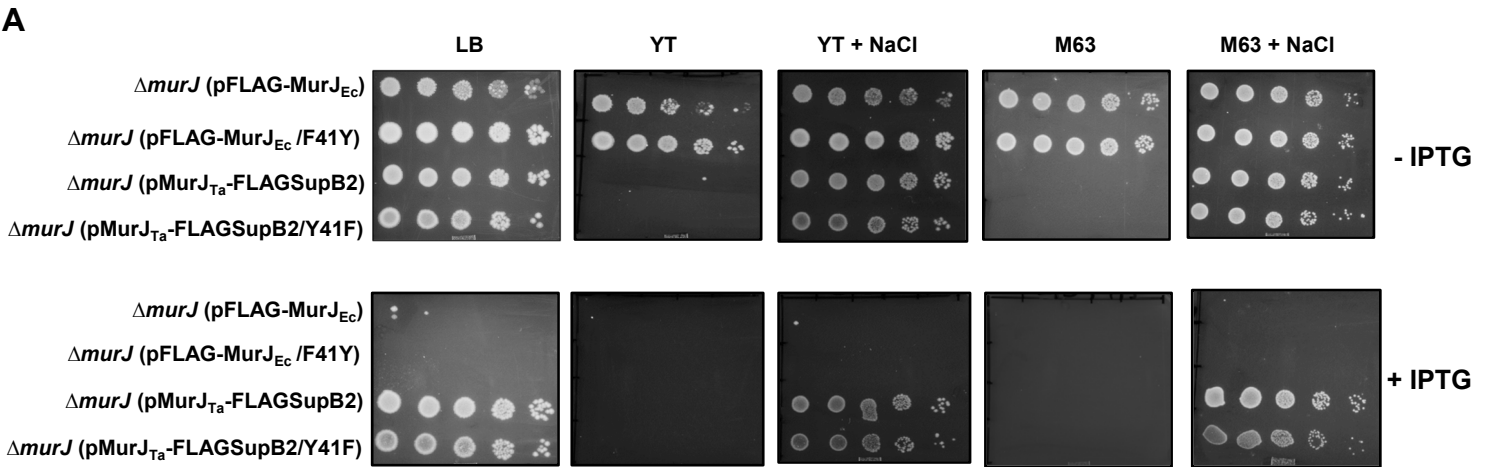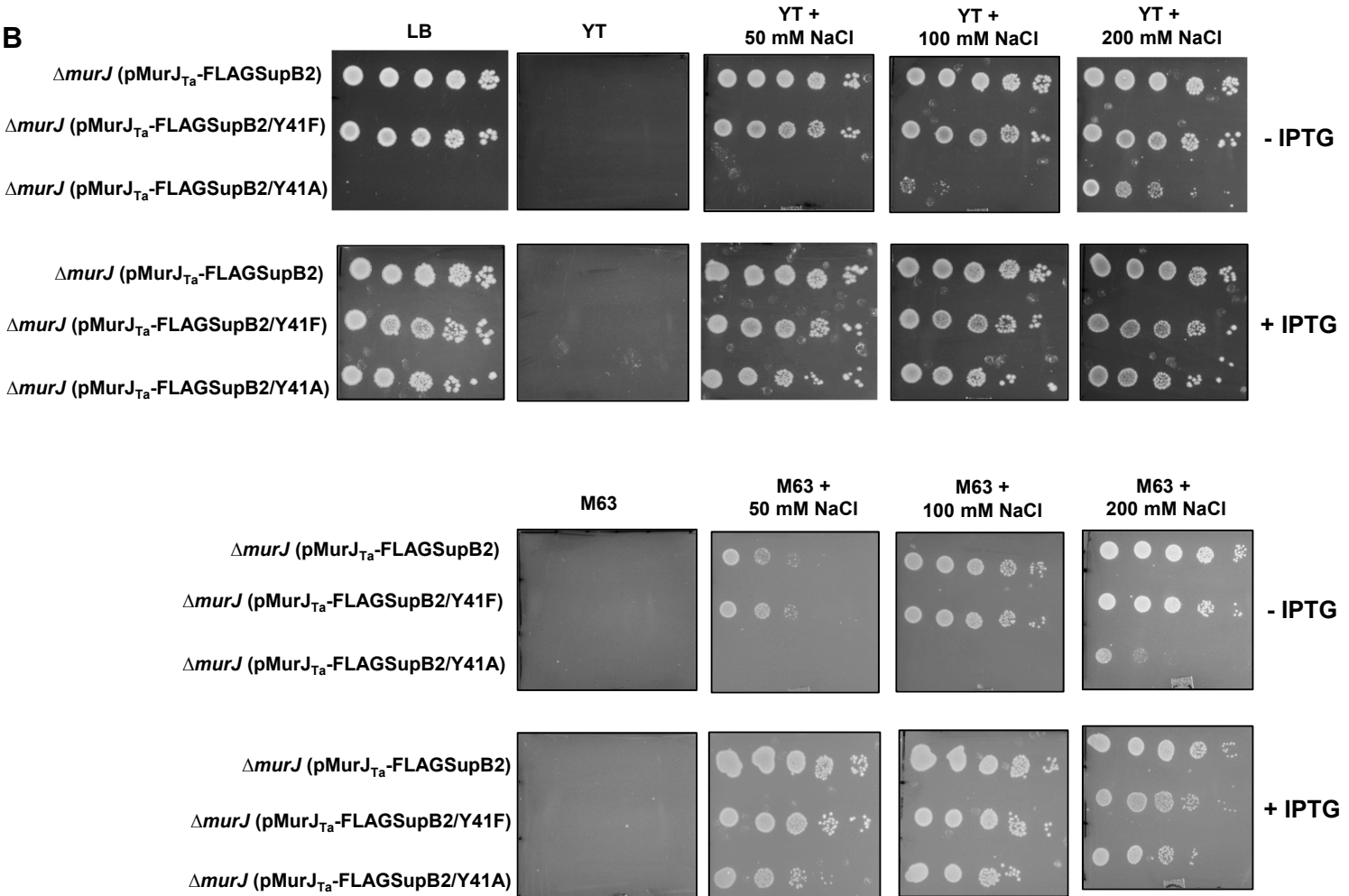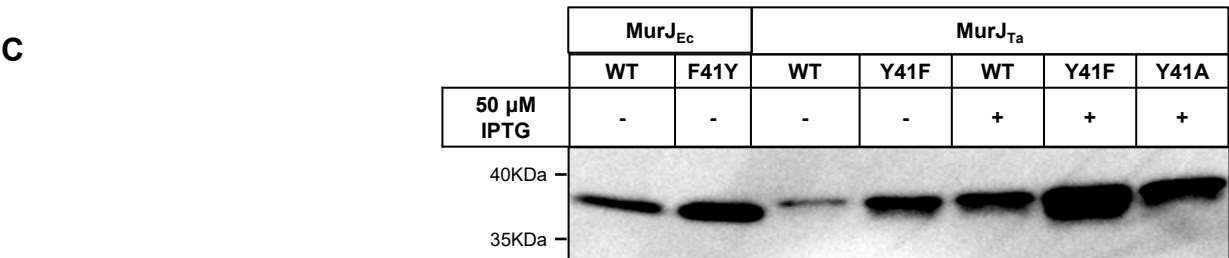

Supplement: FIG S5 [file mbio.00089-23-s0005.pdf]

**A**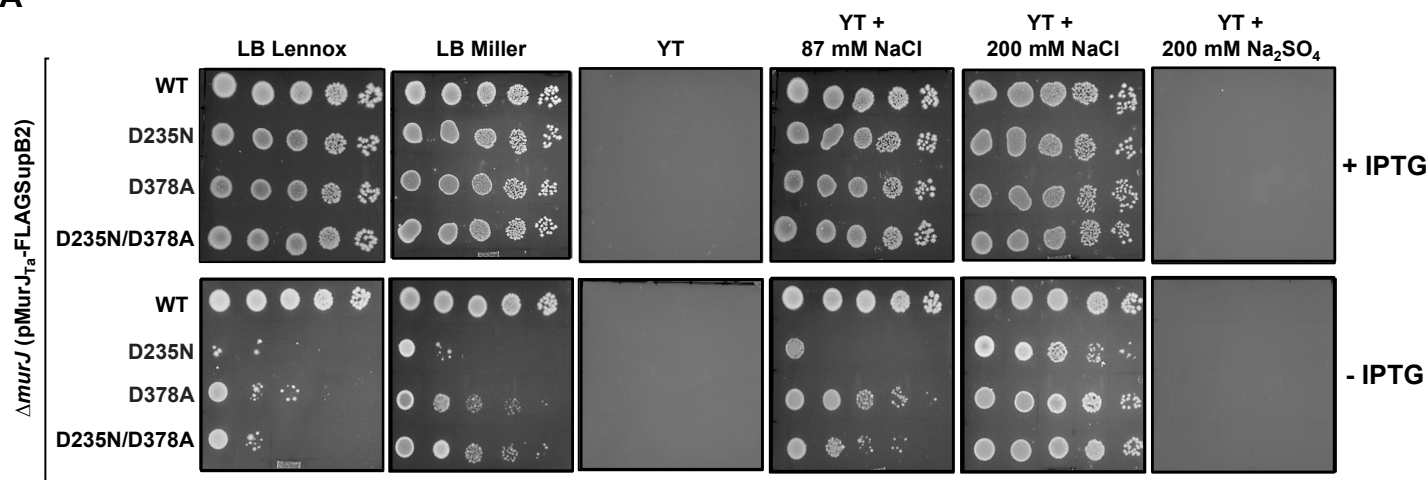**B**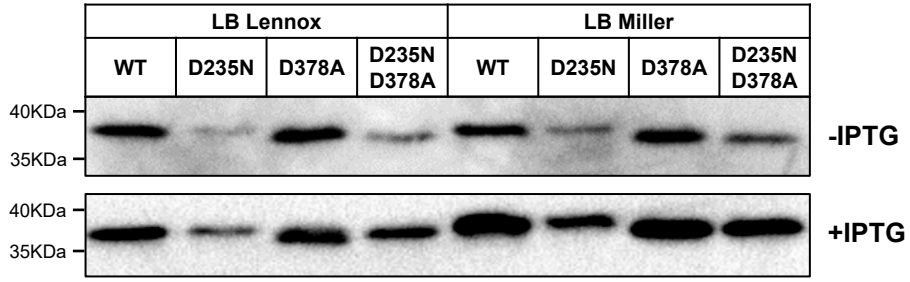**C**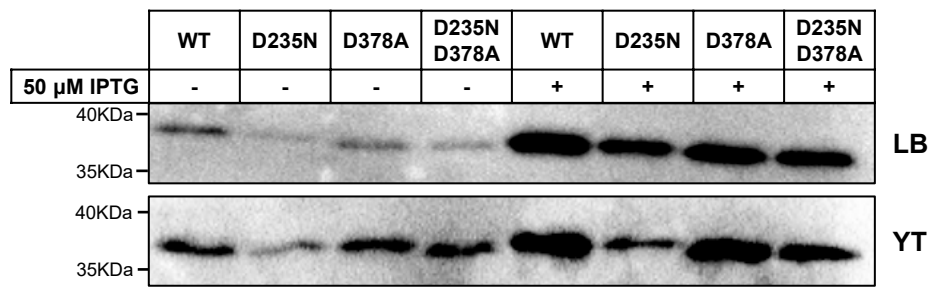

Supplement: FIG S2 [file mbio.00089-23-s0002.pdf]

$\Delta murJ$  (pMurJ<sub>Ta</sub>-FLAGSupB2)

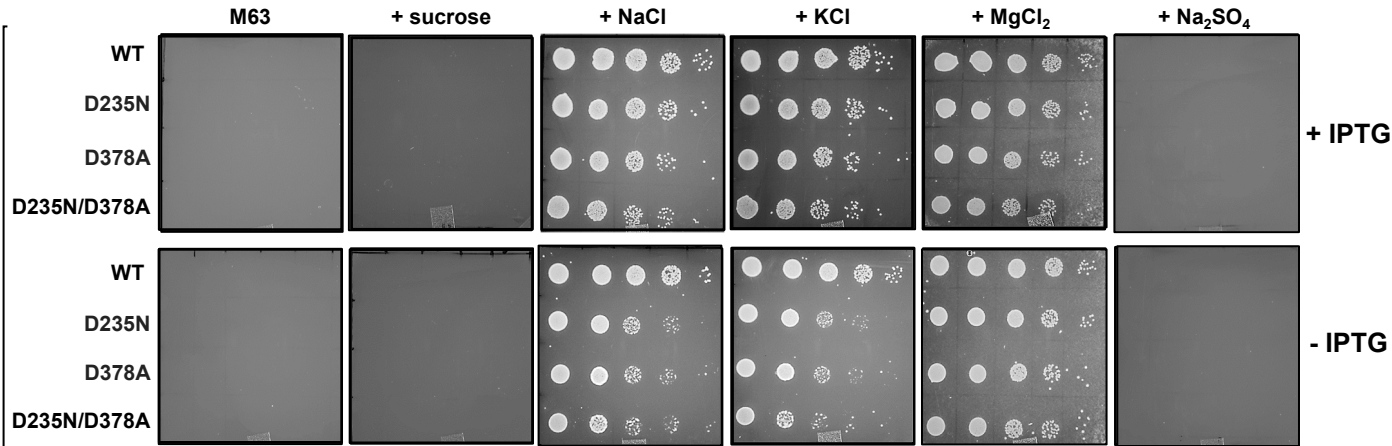

Supplement: FIG S3 [file mbio.00089-23-s0003.pdf]
