# Supplementary material for: Chloride Ions Are Required for Thermosipho africanus MurJ Function
Source: mBio. 2023 Feb 8;14(1):e00089-23. doi: 10.1128/mbio.00089-23 (PMC9973255; doi:10.1128/mbio.00089-23)
Supplement: TABLE S2 [file mbio.00089-23-s0007.pdf]

Table S2: Strains used in this study

Strain	Genotype	Source[a]
Nova Blue	*endA1 hsdR17* (r_{K12}^- m_{K12}^+) *supE44 thi-1 recA1 gyrA96 relA1 lac* F'[*proA*$^+$*B*$^+$ *lacI*^{q}*Z*Δ*M15*::Tn*10*] (Tet^R)	Novagen
MC4100	F$^-$ *araD139* Δ(*argF-lac*) *U169 rpsL150 relA1 flbB5301 deoC1 ptsF25 rbsR*	(1)
NR754	MC4100 *ara*$^+$	(1)
NR3267	NR754 Δ*murJ*::*frt* (pRC7KanMurJ$_{Ec}$)	(2)
NR5191	NR754 Δ*murJ*::*frt* (pFLAGMurJ$_{Ec}$)	This study
FR184	NR754 Δ*murJ*::*frt* (pMurJ$_{Ta}$)	This study
NR5895	NR754 Δ*murJ*::*frt* (pMurJ$_{Ta}$-FLAG)	This study
NR5996	NR754 Δ*murJ*::*frt* (pMurJ$_{Ta}$-FLAGSupB2)	This study
NR6219	NR754 (pMurJ$_{Ta}$-FlagSupB2)	This study
NR7491	NR754 Δ*murJ*::*frt* (pMurJ$_{Ta}$-FLAGSupB2/D235N)	This study
NR7492	NR754 Δ*murJ*::*frt* (pMurJ$_{Ta}$-FLAGSupB2/D378A)	This study
NR7493	NR754 Δ*murJ*::*frt* (pMurJ$_{Ta}$-FLAGSupB2/D235N/D378A)	This study
NR7494	NR754 (pMurJ$_{Ta}$-FLAGSupB2/D235N)	This study
NR7495	NR754 (pMurJ$_{Ta}$-FLAGSupB2/D378A)	This study
NR7496	NR754 (pMurJ$_{Ta}$-FLAGSupB2/D235N/D378A)	This study
NR7739	NR754 Δ*murJ*::*frt* (pFLAGMurJ$_{Ec}$ /F41Y)	This study
NR7740	NR754 Δ*murJ*::*frt* (pMurJ$_{Ta}$-FLAGSupB2/Y41F)	This study
NR7748	NR754 Δ*murJ*::*frt* (pMurJ$_{Ta}$-FLAGSupB2/Y41A)	This study

[a]References:

1. Ruiz N, Wu T, Kahne D, Silhavy TJ. 2006. Probing the barrier function of the outer membrane with chemical conditionality. ACS Chem Biol 1:385-95.
2. Kumar S, Rubino FA, Mendoza AG, Ruiz N. 2019. The bacterial lipid II flippase MurJ functions by an alternating-access mechanism. J Biol Chem 294:981-990.
