# Supplementary material for: Chloride Ions Are Required for Thermosipho africanus MurJ Function
Source: mBio. 2023 Feb 8;14(1):e00089-23. doi: 10.1128/mbio.00089-23 (PMC9973255; doi:10.1128/mbio.00089-23)
Supplement: TABLE S3 [file mbio.00089-23-s0008.pdf]

Table S3: Primers used in this study

Purpose	Primers	Sequence (5' to 3')
Cloning	pFR179 vector fwd	cgcaaaaaataagggtcgacctgcagcc
	pFR179 vector rev	taagatactcatagttaatttctcctctttaatga
	pFR179 insert fwd	gagaaattaactatgagtatcttattcagctcaatcttgt
	pFR179 insert rev	cgacccttattttttgcgggcgtataaaaagc
FLAG insertion	oFR305	ccgcaaaaaaggttctggttctgactacaaagacgacgacgacaaataagggtcgacctgcagc
	oFR306	ggtcgacccttatttgtcgtcgtcgtctttgtagtcagaaccagaaccttttttgcgggcg
SDM	D235N fwd	ccgtagtaaacatgaatgttgtaagtttctacgataag
	D235N rev	cattcatgtttactacggtgttaatttgactcacaac
	D378A fwd	cattttagccattatctttggtctgaaatacggtcc
	D378A rev	caaagataatggctaaaatgatattcgataatgag
	Y41F fwd	tagacgcattctttatcgccatcatgtttccattttttc
	Y41F rev	gcgataaagaatgcgtctaattcataagacacgcc
	Y41A fwd	tagacgcagcctttatcgccatcatgtttccattttttc
	Y41A rev	gcgataaaggctgcgtctaattcataagacacgcc
	EcMurJ F41Y fwd	ccgacgcctatttcgtcgcttttaaacttcctaacttg
	EcMurJ F41Y rev	gcgacgaaataggcgtcggttgccatccctgcgccaaag
